# RESILIENCE AND VULNERABILITY IN GERIATRIC MEDICAL CENTERS DURING AN ONGOING HEALTH CRISIS

**DOI:** 10.1093/geroni/igad104.2856

**Published:** 2023-12-21

**Authors:** Rinat Lifshitz, Irit Asher, Aya Ben Eliezer, Tal Prager Geller, Eduard Zalyesov, Inna Shugaev, Gad Mendelson, Adi Sason

**Affiliations:** The Max Stern Academic College of Emek Yezreel, Israel, Yezreel Valley, HaZafon, Israel; Dorot Medical Center for Rehabilitation and Geriatrics, Netanya, HaZafon, Israel; Fliman Geriatric Hospital, Haifa, HaZafon, Israel; Dorot Care Facility, Netanya, Netanya, HaZafon, Israel; Fliman Medical Center, Haifa, Haifa, HaZafon, Israel; Fliman Geriatric Hospital, Haifa, HaZafon, Israel; Dorot Medical Center for Rehabilitation and Geriatrics, Netanya, HaZafon, Israel; Dorot Geriatric and Rehabilitation Hospital, Netanya, HaZafon, Israel

## Abstract

Coping in stressful times and under cumulative adversities (e.g., health, terror) may lead to emerging vulnerability, diminish resilience and negative psychological consequences. In an organizational framework, this personal vulnerability may also have an impact on organizational resilience. This study examined the resilience and vulnerability indicators during the COVID-19 pandemic and other crises among employees at Geriatric Medical Centers in Israel. Data were collected from a convenience sample of 283 employees from two Geriatric medical centers. The questionnaire included background variables, resilience indicators (personal resilience, morale, wellbeing and a sense of security at home) as well as vulnerability indicators (sense of danger, fear of death and fear of dying, a sense of stress and perception of threats). Administrative workers reported higher vulnerability and lower resilience than workers in other sectors. Longer period of time working with Covid-19 patients was associated with higher resilience and higher vulnerability. After controlling for personal background, death anxiety, sense of danger and health wellbeing significantly correlated with high stress while functional wellbeing and personal morale significantly correlated with low stress. Wellbeing in the workplace was the only indicator associated with high resilience. These findings demonstrate the dialectical experience of employees in geriatrics centers coping with cumulative adversities, combining both vulnerability and resilience. It is recommended for organizations to assist employees in building resilience mechanisms that will help them cope with stressful situations through strengthening their sense of wellbeing, and as a result will help strengthen organizational resilience.

